# Serum metabolomics differentiating pancreatic cancer from new-onset diabetes

**DOI:** 10.18632/oncotarget.16249

**Published:** 2017-03-16

**Authors:** Xiangyi He, Jie Zhong, Shuwei Wang, Yufen Zhou, Lei Wang, Yongping Zhang, Yaozong Yuan

**Affiliations:** ^1^ Department of Gastroenterology, Ruijin Hospital, Shanghai Jiaotong University School of Medicine, Shanghai, China; ^2^ Department of Anesthesia, Ruijin Hospital, Shanghai Jiaotong University School of Medicine, Shanghai, China; ^3^ Department of Gastroenterology, Ruijin Hospital North, Shanghai Jiaotong University School of Medicine, Shanghai, China

**Keywords:** pancreatic cancer, metabolomics, serum, new-onset diabetes

## Abstract

To establish a screening strategy for pancreatic cancer (PC) based on new-onset diabetic mellitus (NO-DM), serum metabolomics analysis and a search for the metabolic pathways associated with PC related DM were performed. Serum samples from patients with NO-DM (n = 30) and patients with pancreatic cancer and NO-DM were examined by liquid chromatography-mass spectrometry. Data were analyzed using principal components analysis (PCA) and orthogonal projection to latent structures (OPLS) of the most significant metabolites. The diagnostic model was constructed using logistic regression analysis. Metabolic pathways were analyzed using the web-based tool MetPA. PC patients with NO-DM were older and had a lower BMI and shorter duration of DM than those with NO-DM. The metabolomic profiles of patients with PC and NO-DM were significantly different from those of patients with NO-DM in the PCA and OPLS models. Sixty two differential metabolites were identified by the OPLS model. The logistic regression model using a panel of two metabolites including N_Succinyl_L_diaminopimelic_acid and PE (18:2) had high sensitivity (93.3%) and specificity (93.1%) for PC. The top three metabolic pathways associated with PC related DM were valine, leucine and isoleucine biosynthesis and degradation, primary bile acid biosynthesis, and sphingolipid metabolism. In conclusion, screening for PC based on NO-DM using serum metabolomics in combination with clinic characteristics and CA19-9 is a potential useful strategy. Several metabolic pathways differed between PC related DM and type 2 DM.

## INTRODUCTION

Pancreatic cancer (PC) is characterized by rapid tumor progression and early metastasis, and is one of the leading causes of cancer-related death. Although the only curative treatment for pancreatic cancer is surgical resection, more than 80% of patients with pancreatic cancer have locally advanced or metastatic tumors that are unresectable at the time of diagnosis [1–3]. Because of the lack of effective early diagnostic methods, there is currently no standard protocol for screening patients at risk of pancreatic cancer (e.g., those with a family history of PC and chronic pancreatitis) [3, 4]. There is increasing evidence that diabetes mellitus (DM) is related to PC [4–7]. Although long-standing diabetes is an etiological factor for PC, new-onset DM (NO-DM) is its manifestation [5–7]. Our group and others proposed a NO-DM based screening strategy for PC [5, 8].

In the last decade, metabolomics has been applied to identify metabolic alterations in various cancers including PC [9]. Diabetes is a complex metabolic disorder. PC related NO-DM, also known as a type-3 DM, may have different metabolic alterations from type-2 NO-DM. To establish a screening strategy for PC based on NO-DM and search for the metabolic pathways associated with PC related type-3 DM, we compared the serum metabolomic profiles of patients with NO-DM and those with PC and NO-DM by liquid chromatography-mass spectrometry (LC–MS).

## RESULTS

The characteristics of the patients included in the analysis are provided in Table 1. The gender distribution and Fasting Blood Glucose were similar between the two groups, whereas the average age differed significantly (P = 0.01), as patients in the NO-DM group were younger than those in the PC with NODM group. The body mass index (BMI) of the PC group was lower than that of the NO-DM group, and the duration of DM was also shorter in the PC group. All patients in the PC group had pancreatic ductal adenocarcinoma (PDAC); however, samples were collected before surgery. The rate of insulin or drug administration was similar between the two groups.

**Table 1:** Characteristics of the study subjects

	PC with new-onset DM	New-onset DM	P value
N	30	30	
Gender			1.0
male	19	20	
female	11	10	
Age, y	62.85±9.86	52.76±9.94	0.001
Stage			
I	3		
II	9		
III	3		
IV	15		
Location			
Head	17		
Tail & body	13		
Differentiation			
I	0		
II	10		
III	20		
BMI (mean± SD)	21.63±3.03	24.77±3.80	0.001
Duration of DM (Mon, mean± SD)	7.2±11.89	16.89±9.65	0.001
CA19-9 (U/L, median, range)	211.5(20-9701)	8.7(0.08-31.5)	0.007
FBG (mmoL/L, mean±SD)	7.65±1.99	7.94±3.92	0.688

**Figure 1:**
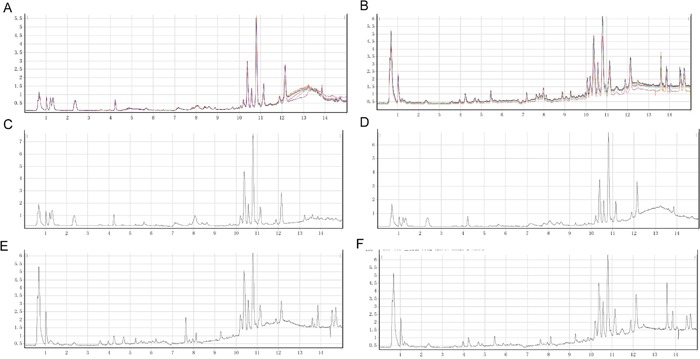
Typical LC/Q-TOF MS total ion chromatograms (TIC) in positive ion mode from serum samples of the Quality Control (QC) sample **(A)**, pancreatic cancer (PC) with diabetes mellitus (DM) patients **(C)**, and a DM control **(E)**. Typical LC/Q-TOF MS TIC chromatograms in negative ion mode from serum samples of the QC sample **(B)**, PC with DM patients **(D)**, and a DM control **(F)**.

**Figure 2:**
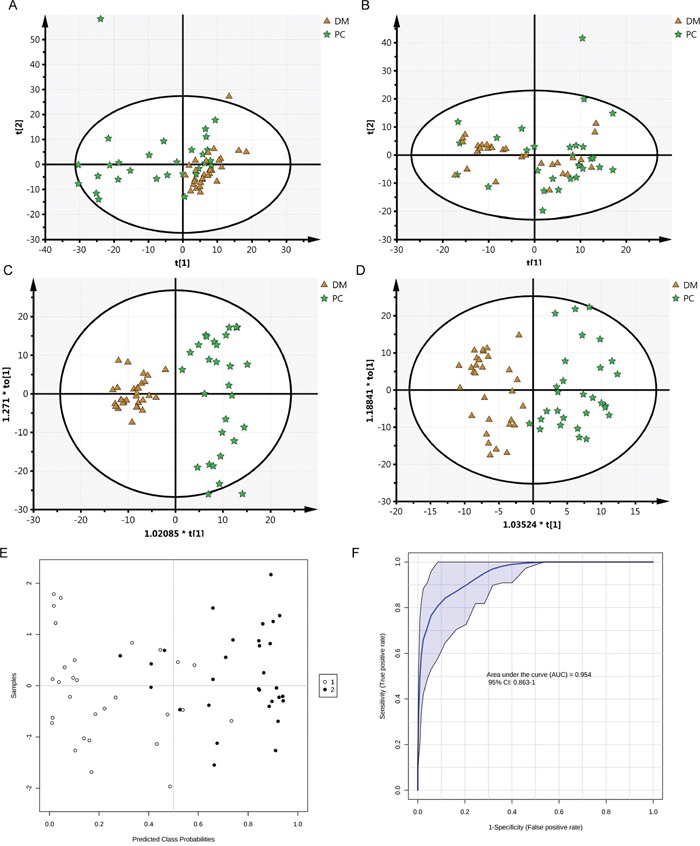
Cluster score plots of unsupervised PCA and supervised OPLS of all patients with PC and DM control DM is represented by circles and malignant samples are depicted as squares. Metabolic profiles depicted by PCA score plots of LC-MS spectral data in negative mode **(A)** or positive mode **(B)**. Metabolic profiles depicted by OPLS score plots of LC-MS spectral data in negative mode **(C)** or positive mode **(D)**. Metabolic profiles depicted by RF score plots of LC-MS spectral data (**E**, 1 PC, 2 DM) and The ROC curves of RF model **(F)**.

To determine the analytical robustness of LC-MS-based methods for global serum metabolic profiling, a pooled quality control (QC) sample was repeatedly analyzed during sample runs. The overlapped total ion current (TIC) chromatograms of the QC sample in both negative and positive modes (Figure 1A and 1B) demonstrated the strong repeatability/reproducibility of our LC-MS system. Typical TIC chromatograms of the serum metabolic profiles of the PC with DM and DM patients analyzed using LC-MS in the positive or negative ion mode are shown in Figure 1C–1F. In the LC-MS dataset, 1577 features were obtained in the negative mode and 873 features in the positive mode. To determine whether the serum metabolic profiles of PC with DM patients were different from those of DM patients, multivariate statistical analysis using the PCA model was performed on the LC-MS spectra of serum samples. The score plots derived from the PCA model are shown in Figure 2A and 2B. Both in the positive mode and negative mode, the R^2^X values of PCA analysis were >0.4, (0.468 and 0.424, respectively). This demonstrated a clear discrimination between the metabolomes of the PC and DM group and the DM group. One case of PC with DM showing an outlier degree was excluded from further analyses. To identify the metabolites that could discriminate between the two groups, a supervised orthogonal projection to latent structures (OPLS) model was built. The score plot showed an obvious separation of PC patients from the DM group (Figure 2C and 2D). The parameters of the OPLS model were R^2^X = 0.172, R^2^Y = 0.816, and Q^2^ = 0.468 in the positive LC-MS mode, and R^2^X = 0.129, R^2^Y = 0.898, and R^2^Y =0.556 in the negative mode. This indicates that these are reliable and predictable models to discriminate between the two groups, as the R^2^Y and R^2^Y values were >0.4. Based on the following two criteria: variable importance in the projection (VIP) value in the OPLS model >1 and *P* value in Student's t test >0.05, 21 variables were identified between the two groups in the positive mode and 41 in the negative mode. Differential metabolites identified in a search of the database (http://metlin.scripps.edu/) are listed in Supplementary Table 1 We next used Random Forest (RF) to build a classification model based on these metabolites. There was a visible separation in the score plots from patients with PC and DM (Figure 2E). The ROC curve based on the probabilities of the RF model resulted in an area under the curve (AUC) of 0.954 [95% confidence interval (CI), 0.863–1.0; Figure 2F].

**Figure 3:**
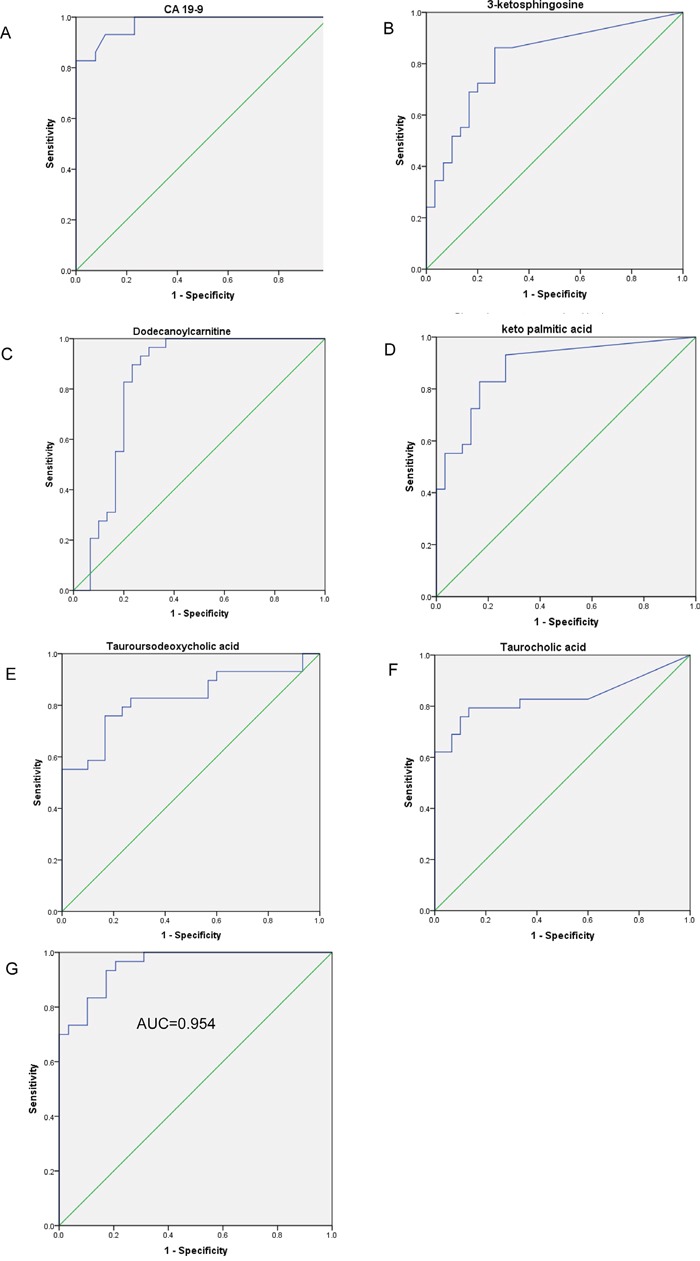
The ROC curves of the logistic regression model and five serum metabolites **(A)** The ROC curves of CA19-9. ROC curves for five metabolites with the highest AUROC: **(B)** 3-ketosphingosine (AUC=0.818, P<0.001, 95% CI 0.708–0.928), **(C)** dodecanoylcarnitine (AUC=0.831, P<0.001, 95% CI 0.714-0.948), **(D)** keto_palmitic_acid (AUC=0.885, P<0.001, 95% CI 0.799–0.971), **(E)** tauroursodeoxycholic_acid (AUC=0.826, P<0.001, 95% CI 0.720–0.949), **(F)** taurocholic_acid (AUC=0.834, P<0.001, 95% CI 0.716–0.937). **(G)** ROC curve analysis for the predictive power of combined plasma biomarkers for distinguishing PC from DM controls. The final logistic model included 2 plasma biomarkers (shown in Table 2).

The AUC of the 62 serum metabolites and CA19-9 were calculated to evaluate their diagnostic performance as individual biomarkers of PC. The ROC curve based on CA19-9 resulted in an AUC of 0.975 (95% CI, 0.943–1.0; Figure 3A); at the cutoff value of 35 U/L the sensitivity and specificity were 82.8% and 100%, respectively. Five metabolites had AUC values of >0.8 (Figure 3B–3F): dodecanoyl carnitine (AUC = 0.831, P < 0.001), 3-ketosphingosine (AUC = 0.818), keto palmitic acid (AUC = 0.885, P < 0.001), taurocholic acid (AUC = 0.834, P < 0.001), and tauroursodeoxycholic acid (AUC = 0.826, P < 0.001). Because the differences in age, BMI, and duration of DM may contribute to the differences in the serum profile, we evaluated the correlation between the levels of the five serum metabolites and three clinical characteristics (Supplementary Table 2). Dodecanoylcarnitine, keto palmitic acid and tauroursodeoxycholic acid showed no relation to age, BMI, and duration of DM.

To construct a more effective diagnostic model for PC, multivariate logistic regression analysis was performed for all the metabolites considering VIP >2.0, age, BMI, and duration of DM. Forward stepwise analysis identified two metabolites among the 62 metabolites, namely PE(18:2) and N_Succinyl_L_diaminopimelic_acid. The parameters of this regression model are shown in Table 2. Our ROC analyses using the predicted possibility of this model revealed an AUC value of 0.951 (95% CI, 0.894−1.000) (Figure 3G). Using a cutoff value of 1, the model's sensitivity and specificity values were 93.3% and 93.1%, respectively.

**Table 2:** Logistic regression analysis of PC-associated plasma metabolite signatures

variables	B	S.E.	Wald	Sig.	Exp(B)
N_Succinyl_L_diaminopimelic_acid	.061	.018	11.434	.001	1.063
PE(18:2)	.013	.005	7.169	.007	1.013
Constant	−5.838	1.767	10.910	.001	.003

**Figure 4:**
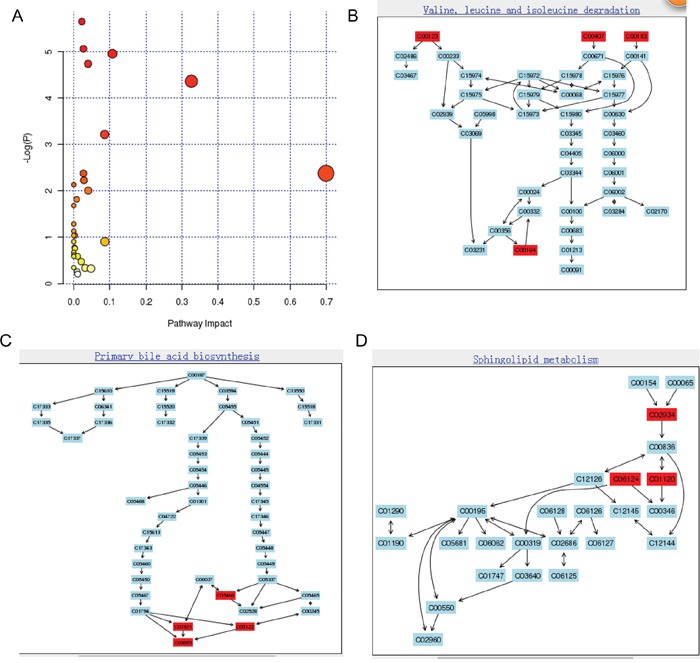
Construction of the altered metabolism pathways in human gastric cancer using MetPA analysis **(A)** Summary of Pathway Analysis. The metabolome view shows all matched pathways according to the P values from pathway enrichment analysis and pathway impact values from pathway topology analysis. Pathway view of involved pathways with the highest original P value calculated from the enrichment analysis: **(B)** valine, leucine, and isoleucine degradation (metabolites marked with red color are C00164/acetoacetic acid, C00123/leucine, C00407/isoleucine, C00183/valine); **(C)** sphingolipid metabolism (metabolites marked with red color are C01921/glycocholic acid, C00695/cholic acid, C05466/chenodeoxycholic acid glycine conjugate, C05122/taurocholic acid); **(D)** primary bile acid biosynthesis (metabolites marked with red color are C02934/3-keto-sphingosine, C06124/C16 sphingosine-1-phosphate, C01120/sphingosine-1-phosphate).

To uncover the metabolic pathways that may participate in PC development, we performed pathway analysis by searching the differential metabolites in the MetPA website. MetPA assigned a total of feature compounds in 26 pathways, which were identified as important for the host response to PC (Figure 4A and Supplementary Table 3). The main metabolic pathways associated with PC or type-3 DM included valine, leucine and isoleucine biosynthesis and degradation (Figure 4B); primary bile acid biosynthesis (Figure 4C); sphingolipid metabolism (Figure 4D); D-glutamine and D-glutamate metabolism; and citrate cycle, as shown in Figure 4 and Supplementary Tables 3 and 4.

## DISCUSSION

Because of the low incidence of PDAC in the general population, population-based screening is not recommended. It is more practical to screen individuals at high risk for PDAC. Increasing evidence suggests that NO-DM is an early manifestation of PC and could be used to improve the detection of asymptomatic, early-stage PC [4]. Therefore, to establish a feasible PC screening strategy based on NO-DM, we compared the serum metabolomic profiles of patients with diabetes and PC with diabetes. Our results showed that the serum metabolomic profiles differed between the two groups, with 62 differential metabolites identified.

PDAC patients with NO-DM were older, and had a lower BMI and shorter duration of DM in our study than those with NO-DM. This is consistent with the results of previous prospective studies. Munigala et al [10]. reported that the risk of PC was higher among patients with NO-DM who were non-obese (relative risk, RR = 1.51) and older than 65 years (RR = 2.01). These factors were to some extent influenced by certain metabolites, as determined by the correlation between the factors and the concentrations of the identified metabolites (data not shown). Three metabolites (AUC >0.8) were not correlated with age, BMI, and duration of DM. This may indicate that the differences in age/BMI/duration of DM may not contribute to the differences in the concentration of these metabolites. Using logistic regression model also balanced the effects of age, BMI, and duration of DM.

Serum metabolomics has been used as a diagnostic tool for PC in a number of recent studies [11–14]. In these studies, three methods have been used, including flow-injection Fourier transform ion cyclotron resonance mass spectrometry (FI-FTICR-MS), ^1^H nuclear magnetic resonance (NMR), and gas chromatography+ mass spectrometry (GC-MS). Compared with the limited sensitivity of 1H NMR and extensive sample preparation (e.g., derivatization) and the low throughput for GC-MS, the high sensitivity and ease of sample preparation in LC-MS make it one the most widely used platforms in metabolomics. Because of the different metabolite coverage detected by different methods, the results generated by different platforms show wide variation in previous studies. By contrast, the metabolic profiles of the present study and those of other reports overlapped [15]. Ritchie et al. [14] performed FI-FTICR-MS metabolomics analysis and showed significant reductions in the serum levels of metabolites belonging to five systems in PC patients compared with controls. The metabolic systems included metabolites belonging to 36-carbon ultra long-chain fatty acids, multiple choline-related systems including phosphatidylcholines, lysophosphatidylcholines, and sphingomyelins, as well as vinyl ether-containing plasmalogen ethanolamines. In the present study, we used a similar system and showed that metabolites belonging to primary bile acid biosynthesis and sphingolipid metabolism are involved in PC. Amino acid-based metabolites including valine, leucine, and isoleucine were identified by our study and two others [12, 16] as discriminating factors of PC from the control group. Our group and that of Bathe et al. [11] showed that the serum concentrations of creatine and glutamine in patients with PC were significantly different from those of patients with benign disease. This indicates that our results are reliable. Unlike previous studies using samples from healthy volunteers as controls, we used NO-DM patients as controls. DM is a possible confounding factor in the PC group. Using NO-DM patients as the control may balance the impact of DM on PC discrimination. Indeed, we were successful in demonstrating a serum metabolomic profile that reflects the presence of PC in NO-DM patients.

Alterations in tumor cell and systemic metabolism are central to the biology of PC [7]. PC causes changes in whole-body metabolism, and results in alterations in circulating metabolites. Therefore, investigation of PC associated metabolic pathways provides important insight into how these tumors develop and grow, and suggests new approaches for its detection, prevention, and treatment. Our results showed that the altered circulating metabolites belonged to the following metabolic pathways: valine, leucine, and isoleucine biosynthesis and degradation, D-glutamine and D-glutamate metabolism, primary bile acid biosynthesis, sphingolipid metabolism, and citrate cycle. The analyses of sera from PDAC patients often shows disturbed amino acid profiles compared with those of healthy individuals [7]. Total serum amino acid concentration is reduced in PC patients [17]. Patients with PC often show malnutrition because of pancreatic endocrine and exocrine insufficiency [13]. Mayers et al. [18] showed that elevated plasma levels of all three proteinogenic essential branched chain amino acids (BCAAs) (isoleucine, leucine, and valine) are associated with the diagnosis of PDAC. By contrast, our group and others showed that plasma BCAAs are decreased in patients with PC compared with controls. This may indicate that when cancer has already developed, amino acid exhaustion results in the reduction of these amino acids. Glucose and glutamine metabolism is one of the most disturbed pathways in PC and has been intensively investigated for its potential therapeutic value [7, 19, 20]. Glutamine-dependent metabolism is critical for PC cell growth [21]. However, whether the metabolic rewiring of PDAC is attributed to changes of serum metabolite levels in PDAC is unknown. It is well recognized that bile secretion and cholic acid biosynthesis are disturbed in PC. However, whether the increase of serum bile acids or their derivatives is one of the causes of PC remains unclear. The value of serum bile acids or their derivatives as biomarkers of PDAC may be limited because they may be increased in hepatic diseases and could be influenced by dietary intake. Our results showed that cholic acid and deoxycholic acid were decreased in PDAC, whereas taurocholic acid and glycocholic acid were increased. Therefore, the ratio of different components of cholic acid is an important consideration.

Although our approach to diagnosing pancreatic cancer is novel and promising, there were some limitations to this study. Firstly, the sample size was small and the rate of early stage disease was low. Secondly, LC-MS may not be suitable for screening large populations because of its high cost in developing countries. Further study is needed to validate and optimize cost-effective methods for the detection of the identified serum metabolic biomarkers. Thirdly the difference in age and BMI may also contribute to the difference in the serum profile. Fourthly the AUC of our regression model was not higher than CA19-9, but the sensitivity is higher than CA19-9. Our serum metabolites’ regression model could be used as compensation for CA19-9.

In summary, we demonstrated that it is possible to distinguish the metabolomic profile of PC from that of patients with new-onset diabetes in sera. Our future work will involve the refinement of the metabolomic profile obtained, as well as the identification of a more comprehensive profile. Plasma metabolic signatures show promise as biomarkers for the early detection of PC, and the identification of altered metabolic pathways between type-2 diabetes and PC related type-3 diabetes may help understand the mechanisms underlying the association between PC and diabetes.

## MATERIALS AND METHODS

### Sample collection

Blood samples from PC with NO-DM (less than 2 year) patients and 30 NO-DM (less than 2 year) treated in our hospital at the same time were collected and stored at −80°C until analysis. The patients with NO-DM were followed for an additional 2 years or more and none of them developed PC. All samples were collected before surgery. The study was approved by the ethics committee of Ruijin Hospital, Shanghai Jiaotong University School of Medicine and all subjects signed informed consent forms.

### LC-MS

Serum samples were thawed and proteins were precipitated by adding 300 mL of cold methanol to 100 mL of serum. The mixture was then centrifuged at 12,000 g for 30 min and the supernatant was stored for further analysis. LC-MS analysis was performed using an Agilent LC-Q/TOF-MS system (Agilent Technologies, Santa Clara, CA, USA) consisting of an Agilent 1290 liquid chromatography system coupled online with an Agilent 6530 time-of-flight mass spectrometer. A 4 μL aliquot of each sample was injected onto a 2.1 × 100 mm Agilent C18 1.8 μm particle column, heated to 40°C, gradient-eluted at 0.4 mL/min using mobile phase A (0.1% acetic acid in water) and mobile phase B (0.1% acetic acid in acetonitrile). The gradient of the mobile phase is shown in Supplementary Table 5. Electrospray ionization was used in both negative mode and positive mode. The following parameters were used for the negative mode: capillary voltage; 3.5 kV, sampling cone, 50 kV; source temperature, 100°C; desolvation temperature 300°C, cone gas flow, 50 L/h; desolvation gas flow, 700 L/h; extraction cone, 4 V; scan time, 0.03 s; inter scan time, 0.02 s; data acquisition region, 50–1000 m/z. The following parameters were used for the positive mode: capillary voltage, 4 kV; sampling cone, 35 kV; source temperature, 100°C; desolvation temperature, 350°C; cone gas flow, 50 L/h; desolvation gas flow, 600 L/h; extraction cone, 4 V. To ensure accuracy and repeatability, leucine encephalin was used as lock mass, which generated a 556.2771 Da [M+H]+ ion in positive mode and a 554.2615 Da [M-H]^−^ion in negative mode.

### Statistical analysis

MS data were processed using Mass Profiler software (Agilent), and further subjected to PCA, OPLS, and RF using the SIMCA-P1 11 software package (Umetrics, Umeå, Sweden) for multivariate pattern recognition analysis. OPLS-DA was performed to discriminate between PC and DM patients and DM controls. Metabolites with VIP >1 and p <0.05 were considered to be potential markers capable of differentiating PC from NO-DM controls. Calculation of AUROC and logistic regression was performed using SPSS20 software (IBM SPSS Inc., Chicago, IL, USA). Altered metabolic pathways in PC were analyzed by MetPA, a web-based metabolomics tool for pathway analysis and visualization [22].

## SUPPLEMENTARY MATERIALS TABLES





## Abbreviations

(NO-DM): new-onset diabetic mellitus
(PC): pancreatic cancer
(LC–MS): liquid chromatography-mass spectrometry
(OPLS): orthogonal projection to latent structures
(TIC): total ion current
(RF): Random Forest
(VIP): importance in the projection
(PCA): principal component analysis
