# Supplementary material for: Serum metabolomics differentiating pancreatic cancer from new-onset diabetes
Source: Oncotarget. 2017 Mar 16;8(17):29116–24. doi: 10.18632/oncotarget.16249 (PMC5438717; doi:10.18632/oncotarget.16249)
Supplement: Supplementary file 1 [file oncotarget-08-29116-s001.pdf]

Serum metabolomics differentiating pancreatic cancer from new-onset diabetes

SUPPLEMENTARY TABLES

Supplementary Table 1: Differential plasma metabolites in PC with DM patients compared to controls (DM patients)

See Supplementary File 1

Supplementary Table 2: The P value of nonparametric correlations (Spearman test) between each parameter

	Dodecanoylcarnitine	3-ketosphingosine	keto_ palmitic_acid	Taurocholic_ acid	Tauroursodeoxycholic_ acid
age	0.083	**0.006**	0.016	0.564	0.397
BMI	0.734	0.121	0.230	**0.039**	0.129
Duration of DM	0.186	0.062	0.116	0.146	0.305

Supplementary Table 3: The identified metabolites and their matched pathways analyzed by MetPA

See Supplementary File 1

Supplementary Table 4: Results from pathway analysis with MetPA

Metabolite Set	Total	Expected	Hits	Raw p	Raw p	Holm p	FDR Impact
Valine, leucine and isoleucine degradation	40	0.65	4	3.54E-03	2.83E-01	1.76E-01	0.02
Primary bile acid biosynthesis	47	0.76	4	6.36E-03	5.03E-01	1.76E-01	0.03
Sphingolipid metabolism	25	0.41	3	7.07E-03	5.52E-01	1.76E-01	0.11
Valine, leucine and isoleucine biosynthesis	27	0.44	3	8.79E-03	6.77E-01	1.76E-01	0.04
D-Glutamine and D-glutamatemetabolism	11	0.18	2	1.28E-02	9.75E-01	2.05E-01	0.33
Citrate cycle (TCA cycle)	20	0.32	2	4.05E-02	1.00E+00	5.39E-01	0.09
Synthesis and degradation of ketone bodies	6	0.10	1	9.35E-02	1.00E+00	9.36E-01	0.70
Vitamin B6 metabolism	32	0.52	2	9.36E-02	1.00E+00	9.36E-01	0.03
Propanoate metabolism	35	0.57	2	1.09E-01	1.00E+00	9.57E-01	0.03
Aminoacyl-tRNA biosynthesis	75	1.22	3	1.20E-01	1.00E+00	9.57E-01	0.00
Butanoate metabolism	40	0.65	2	1.36E-01	1.00E+00	9.89E-01	0.04
Ascorbate and aldarate metabolism	45	0.73	2	1.64E-01	1.00E+00	1.00E+00	0.01

Total is the total number of compounds in the pathway; Hits is the matched number from the user uploaded data; Raw p is the original p value calculated from the enrichment analysis; Holm p is the p value adjusted by the Holm-Bonferroni method; FDR p is the p value adjusted using False Discovery Rate; Impact is the pathway impact value calculated from pathway topology analysis.

Supplementary Table 5: Mobile phase gradient

Time(min)	Flow(ml/min)	Pressure Limit(bar)	Solv Ratio B(%)
0	0.35	800	5
1	0.35	800	5
6	0.35	800	20
9	0.35	800	50
13	0.35	800	95
15	0.35	800	95
