# Supplementary material for: Serum metabolomics differentiating pancreatic cancer from new-onset diabetes
Source: Oncotarget. 2017 Mar 16;8(17):29116–24. doi: 10.18632/oncotarget.16249 (PMC5438717; doi:10.18632/oncotarget.16249)
Supplement: Supplementary file 2 [file oncotarget-08-29116-s002.docx]

Table s1. Differential plasma metabolites in PC with DM patients compared to controls (DM patients)

| name | ttest | fold change(PC/DM) | VIP | mass | Mode |
| --- | --- | --- | --- | --- | --- |
| 3-Indoleacetic Acid | 0.016 | 0.63 | 1.396 | 175.0662 | Neg |
| 7-oxo-11-Tetradecenoic acid | 0.006 | 1.69 | 1.570 | 240.1718 | Neg |
| Acetoacetic acid | 0.036 | 1.92 | 1.219 | 102.0327 | Neg |
| C16 Sphingosine-1-phosphate | 0.000 | 0.57 | 2.192 | 351.2167 | Neg |
| Chenodeoxycholic acid glycine conjugate | 0.008 | 12.03 | 1.534 | 449.3139 | Neg |
| Cholic acid | 0.038 | 0.18 | 1.209 | 408.2868 | Neg |
| cis-9-palmitoleic acid | 0.001 | 1.68 | 1.808 | 254.2235 | Neg |
| Deoxycholic acid | 0.002 | 0.41 | 1.744 | 392.2917 | Neg |
| Glycocholic Acid | 0.003 | 80.09 | 1.714 | 465.3089 | Neg |
| HOME | 0.020 | 2.01 | 1.341 | 298.25 | Neg |
| hydroxy capric acid | 0.025 | 1.59 | 1.297 | 188.1408 | Neg |
| hydroxy stearic acid | 0.043 | 1.84 | 1.179 | 300.2646 | Neg |
| hydroxypalmitic acid | 0.004 | 2.15 | 1.661 | 272.2344 | Neg |
| Indoxylsulfuric acid | 0.012 | 0.62 | 1.454 | 213.0091 | Neg |
| kamlolenic acid | 0.034 | 1.93 | 1.231 | 294.2166 | Neg |
| keto myristic acid | 0.004 | 2.05 | 1.647 | 242.1875 | Neg |
| keto palmitic acid | 0.000 | 6.15 | 2.536 | 270.2185 | Neg |
| Ketoleucine | 0.020 | 0.80 | 1.348 | 130.0631 | Neg |
| Isoleucine | 0.000 | 0.76 | 2.260 | 131.0945 | Neg |
| Leucine | 0.001 | 0.80 | 1.890 | 131.0947 | Neg |
| LysoPE(22:5) | 0.021 | 0.75 | 1.336 | 527.3005 | Neg |
| N-Succinyl-L-diaminopimelic acid | 0.000 | 0.19 | 2.880 | 290.1123 | Neg |
| Oleic Acid | 0.005 | 1.80 | 1.595 | 282.2551 | Neg |
| Oxalosuccinic acid | 0.011 | 0.57 | 1.464 | 190.0101 | Neg |
| oxo-decanoic acid | 0.008 | 0.46 | 1.525 | 186.1251 | Neg |
| Oxoglutaric acid | 0.024 | 3.21 | 1.311 | 146.0219 | Neg |
| PC(17:0)/PE(20:0) | 0.001 | 0.71 | 1.822 | 509.3471 | Neg |
| PE(18:1)/PC(15:1) | 0.003 | 0.76 | 1.699 | 479.3005 | Neg |
| PE(18:3) | 0.007 | 0.48 | 1.536 | 475.2689 | Neg |
| PE(20:5) | 0.004 | 0.56 | 1.638 | 499.2691 | Neg |
| PE(22:4) | 0.023 | 0.70 | 1.313 | 529.3157 | Neg |
| Phenyllactic acid | 0.014 | 1.72 | 1.414 | 166.0625 | Neg |
| Sphingosine-1-phosphate | 0.017 | 0.86 | 1.382 | 379.2481 | Neg |
| Taurocholic acid | 0.003 | 747.13 | 1.719 | 515.2912 | Neg |
| Tauroursodeoxycholic acid | 0.003 | 111.35 | 1.678 | 499.2963 | Neg |
| Tetradecanedioic acid | 0.010 | 2.11 | 1.488 | 258.1824 | Neg |
| Threonic acid | 0.009 | 0.72 | 1.506 | 136.0377 | Neg |
| Uric acid | 0.003 | 0.76 | 1.693 | 168.0293 | Neg |
| Uridine | 0.000 | 0.67 | 2.056 | 244.0692 | Neg |
| α-hydroxy lauric acid | 0.024 | 1.81 | 1.312 | 216.172 | Neg |
| α-hydroxy myristic acid | 0.009 | 1.90 | 1.510 | 244.2031 | Neg |
| 3-ketosphingosine | 0.000 | 4.10 | 2.500 | 297.2674 | Pos |
| Arachidonoyl dopamine | 0.000 | 0.48 | 2.319 | 439.3074 | Pos |
| Arachidyl carnitine | 0.033 | 0.62 | 1.321 | 455.3986 | Pos |
| Bilirubin | 0.027 | 1.97 | 1.088 | 584.2649 | Pos |
| Creatine | 0.001 | 0.46 | 1.462 | 131.0695 | Pos |
| Dodecanoylcarnitine | 0.007 | 1.60 | 1.466 | 343.2733 | Pos |
| Elaidic carnitine | 0.044 | 1.29 | 1.252 | 425.3522 | Pos |
| Glutamate | 0.000 | 2.25 | 1.909 | 147.0534 | Pos |
| L-Hexanoylcarnitine | 0.022 | 1.38 | 1.414 | 259.1788 | Pos |
| N-Oleoyl-L-Serine | 0.000 | 5.60 | 1.762 | 369.2891 | Pos |
| N-stearoyl serine | 0.003 | 1.72 | 1.782 | 371.3046 | Pos |
| PC(18:0)/PE(21:0) | 0.045 | 1.61 | 1.242 | 523.3692 | Pos |
| PC(20:5) | 0.007 | 0.77 | 1.202 | 541.3161 | Pos |
| PE(18:0)/PC(15:0) | 0.010 | 0.70 | 1.473 | 481.3184 | Pos |
| PE(18:2) | 0.000 | 0.58 | 2.594 | 477.2868 | Pos |
| PE(20:4) | 0.018 | 0.81 | 1.419 | 501.2878 | Pos |
| Propionyl-L-carnitine | 0.033 | 0.70 | 1.319 | 217.1316 | Pos |
| Pyroglutamic acid | 0.017 | 0.66 | 1.471 | 129.0426 | Pos |
| tetracosahexaenoic acid | 0.012 | 0.44 | 1.549 | 356.2723 | Pos |
| Ubiquinone-1 | 0.000 | 0.38 | 2.939 | 250.121 | Pos |
| Valine | 0.007 | 0.81 | 2.573 | 117.0792 | Pos |

Table s3. The identified metabolites and their matched pathways analyzed by MetPA

| Pathways | Name | Match | HMDB | KEGG |
| --- | --- | --- | --- | --- |
| Valine, leucine and isoleucine degradation | Acetoacetic acid | Acetoacetic acid | HMDB00060 | C00164 |
|  | Leucine | L-Leucine | HMDB00687 | C00123 |
|  | Isoleucine | L-Isoleucine | HMDB00172 | C00407 |
|  | Valine | L-Valine | HMDB00883 | C00183 |
| Primary bile acid biosynthesis | Glycocholic Acid | Glycocholic acid | HMDB00138 | C01921 |
|  | Cholic acid | Cholic acid | HMDB00619 | C00695 |
|  | Chenodeoxycholic acid glycine conjugate | Chenodeoxycholic acid glycine conjugate | HMDB00637 | C05466 |
|  | Taurocholic acid | Taurocholic acid | HMDB00036 | C05122 |
| Sphingolipid metabolism | 3-keto-sphingosine | 3-Dehydrosphinganine | HMDB01480 | C02934 |
|  | C16 Sphingosine-1-phosphate | Sphingosine 1-phosphate | HMDB00277 | C06124 |
|  | Sphingosine-1-phosphate | Sphinganine 1-phosphate | HMDB01383 | C01120 |
| Valine, leucine and isoleucine biosynthesis | Valine | L-Valine | HMDB00883 | C00183 |
|  | Leucine | L-Leucine | HMDB00687 | C00123 |
|  | Isoleucine | L-Isoleucine | HMDB00172 | C00407 |
| D-Glutamine and D-glutamate metabolism | Glutamate | D-Glutamic acid | HMDB03339 | C00217 |
|  | Oxoglutaric acid | Oxoglutaric acid | HMDB00208 | C00026 |
| Citrate cycle (TCA cycle) | Oxalosuccinic acid | Oxalosuccinic acid | HMDB03974 | C05379 |
|  | Oxoglutaric acid | Oxoglutaric acid | HMDB00208 | C00026 |
| Synthesis and degradation of ketone bodies | Acetoacetic acid | Acetoacetic acid | HMDB00060 | C00164 |
| Vitamin B6 metabolism | Oxoglutaric acid | Oxoglutaric acid | HMDB00208 | C00026 |
|  | Glutamate | D-Glutamic acid | HMDB03339 | C00217 |
| Propanoate metabolism | Valine | L-Valine | HMDB00883 | C00183 |
|  | Acetoacetic acid | Acetoacetic acid | HMDB00060 | C00164 |
| Butanoate metabolism | Oxoglutaric acid | Oxoglutaric acid | HMDB00208 | C00026 |
|  | Acetoacetic acid | Acetoacetic acid | HMDB00060 | C00164 |
| Ascorbate and aldarate metabolism | Threonic acid | Threonic acid | HMDB00943 | C01620 |
|  | Oxoglutaric acid | Oxoglutaric acid | HMDB00208 | C00026 |
| Fatty acid biosynthesis | cis-9-palmitoleic acid | Palmitoleic acid | HMDB03229 | C08362 |
|  | Oleic Acid | Oleic acid | HMDB00207 | C00712 |
| Taurine and hypotaurine metabolism | Taurocholic acid | Taurocholic acid | HMDB00036 | C05122 |
| Alanine, aspartate and glutamate metabolism | Oxoglutaric acid | Oxoglutaric acid | HMDB00208 | C00026 |
| Pantothenate and CoA biosynthesis | Valine | L-Valine | HMDB00883 | C00183 |
| Lysine biosynthesis | Oxoglutaric acid | Oxoglutaric acid | HMDB00208 | C00026 |
| Glutathione metabolism | Pyroglutamic acid | Pyroglutamic acid | HMDB00267 | C01879 |
| Glycerophospholipid metabolism | LysoPE(22:5) | LysoPC(22:5(4Z,7Z,10Z,13Z,16Z)) | HMDB10402 | C04230 |
| Histidine metabolism | Oxoglutaric acid | Oxoglutaric acid | HMDB00208 | C00026 |
| Glycine, serine and threonine metabolism | Creatine | Creatine | HMDB00064 | C00300 |
| Glyoxylate and dicarboxylate metabolism | Oxoglutaric acid | Oxoglutaric acid | HMDB00208 | C00026 |
| Tyrosine metabolism | Acetoacetic acid | Acetoacetic acid | HMDB00060 | C00164 |
| Arginine and proline metabolism | Creatine | Creatine | HMDB00064 | C00300 |
| Tryptophan metabolism | 3-Indoleacetic Acid | Indoleacetic acid | HMDB00197 | C00954 |
| Purine metabolism | Uric acid | Uric acid | HMDB00289 | C00366 |
| Porphyrin and chlorophyll metabolism | Bilirubin | Bilirubin | HMDB00054 | C00486 |
